# South African Parents’ and Grandparents’ Perspectives on the Acceptability of Implant Delivery of Treatment to Young Children with HIV

**DOI:** 10.1007/s10461-024-04515-8

**Published:** 2024-10-28

**Authors:** Imogen Hawley, Alejandro Baez, Fiona Scorgie, Lee Fairlie, Florence Mathebula, Mackenzie Leigh Cottrell, Leah M. Johnson, Elizabeth T. Montgomery

**Affiliations:** 1 Women’s Global Health Imperative, RTI International, Berkeley, CA USA; 2 Wits RHI, Faculty of Health Sciences, University of the Witwatersrand, Johannesburg, South Africa; 3 UNC Eshelman School of Pharmacy, Chapel Hill, NC USA; 4 RTI International, Research Triangle Park, NC USA; 5 Department of Epidemiology and Biostatistics, University of California, San Francisco, USA

**Keywords:** HIV treatment, Caregivers, Implants, Pediatric populations, Qualitative end-user research

## Abstract

Children living with HIV (CLWH) face unique challenges with adherence to antiretroviral therapy. In South Africa, just over a third of children receiving antiretroviral therapy are virally suppressed. Long-acting, subcutaneous implants may improve outcomes in CLWH compared to current daily oral dosing regimens. Qualitative in-depth interviews and focus group discussions (FGD) were conducted with 50 caregivers of CLHW in Johannesburg, South Africa. Interviews and FGDs were audio-recorded and transcribed. Data were coded and analyzed using Dedoose v9 software and a thematic approach. Caregivers had generally positive impressions of the pediatric HIV treatment implant. They emphasized the advantages of a long-acting and discreet treatment option for CLWH. Cited advantages were perceived to have widespread impact on CLWH, their caregivers, and other social dynamics. Caregivers raised some concerns or uncertainties about the potential efficacy, side effects and safety of the implant. Future clinical testing and outreach efforts may address such concerns and mitigate potential misinformation about implants. This study indicates the need to develop long-acting, discreet, safe, and efficacious HIV treatment options for young children.

## Introduction

Roughly 1.5 million children aged 0–14 years were living with HIV globally in 2022 [1]. Of those, 87% were living in Eastern and Southern Africa and nearly a quarter million in South Africa alone [2]. Despite advancements in HIV testing and delivery of antiretroviral therapy (ART), only half the children living with HIV (CLWH) in Eastern and Southern Africa, and as few as 37% in South Africa, were virally suppressed [2]. While adherence to ART is critical to the management of HIV, numerous barriers prevent CLWH in this setting from adhering to ART properly. The current pediatric formulations for ART are oral medications, which present hurdles such as poor palatability, complex regimens, and difficulty swallowing, as well as challenges for caregivers when it comes to transporting and storing medications [3–5]. Furthermore, CLWH and their caregivers often face various psychosocial factors that prevent them from adhering to ART, such as stigma, nondisclosure of the child’s HIV status, and lack of medical literacy [6].

Commonly used ART medications for pediatric HIV treatment include abacavir, lamivudine, dolutegravir, lopinavir/ritonavir and in neonates, zidovudine, lamivudine and nevirapine given for approximately one month, before switching to pediatric regimens. Treatment formulations include solid and dispersible tablets, as well as granules and pellets, usually within capsules, which can be crushed or opened and mixed with food to address non-palatability and difficulties swallowing. Syrups are also available in glass containers that may be challenging to store and dose correctly, but can be flavored to make more palatable. While there has been some progress toward resolving the challenges children face in taking these medications, particularly in the youngest children, regimens may still be cumbersome and complicate dosing and adherence. There is a critical need for improved drug delivery mechanisms for children. Evaluation of long-acting injectable cabotegravir/rilpivirine for children is currently under investigation [7], and additional delivery systems, such as microarray patches, are being developed [8]. Simplified, pediatric-friendly ART formulations, dosing regimens and delivery systems, particularly those that are long-acting and administered less frequently to assist with improved adherence, are important priorities for pediatric HIV control to improve the lives of CLWH and their caregivers [9].

Biodegradable implants for pediatric HIV treatment are at the pre-clinical stage of product development, and our research team was uniquely positioned to bridge potential end-user insights with product development efforts. In collaboration with our implant development team, we assessed the acceptability of this novel long-acting approach with parent and grandparent caregivers of young South African children living with HIV. Although it is children who would be using this implant in the future, we refer to caregivers as the product end-users given their decision-making role in the children’s care, their responsibility for current product administration, and because they are the arbitrators of consent to treatment for their children. The goal was to solicit end-user feedback at an early stage, while product design was still modifiable, to improve the implant’s acceptability, use, and public health impact. As potential end-users, caregivers may provide valuable perspectives about the utility of a long-acting subcutaneous implant for pediatric HIV treatment.

## Methods

### Study Design

In this qualitative, cross-sectional study on the Delivery of Antiretrovirals via Implantable System for Young children (DAISY), in-depth interviews (IDIs) and focus group discussions (FGDs) were conducted with caregivers of CLWH aged 2–5 years old living in Johannesburg, South Africa. The DAISY study protocol was approved by the Human Research Ethics Committee (Medical) of the University of the Witwatersrand and Salus, an independent institutional review board in the United States servicing RTI International. The study was overseen by the regulatory infrastructure of the Division of AIDS (DAIDS).

### Sampling and Recruitment

Using quota sampling frames and purposive voluntary sampling, 50 caregivers of CLWH aged 2–5 years old were purposively recruited from selected public sector children’s clinics in Johannesburg, South Africa. Caregivers were defined as being the self-reported primary guardian responsible for administering the child’s HIV treatment. Caregivers 18 years of age or older were eligible for participation if their children, aged 2–5 years old, had been using ART for at least 6 months, and if they were fluent in English, isiZulu, or Sesotho, and were willing and able to provide written informed consent.

### Data Collection

Data collection was conducted in two phases, starting with 16 IDIs to gather initial perspectives on and experiences with the management of HIV treatment for their child (and their family, if applicable) and attitudes toward a hypothetical HIV treatment implant for children. IDI sample quotas of equal size (*n* = 4) were set to assess differences in end-user acceptability by two variables: relationship to their CLWH (i.e., *n* = 8 biological mothers and *n* = 8 fathers or other types of guardians) and ART adherence based on the CLWH’s viral load (i.e., *n* = 8 with low viral load defined as ≤ 1000 copies/mL and *n* = 8 with high viral load defined as > 1000 copies/mL). Then 8 FGDs with 4–5 participants in each group were conducted to elicit group-based questions, debates, concerns, and normative attitudes regarding long-acting pediatric HIV treatment and the development of an implant for this indication. CLWH’s viral load was excluded from FGD selection criteria due to difficulty finding caregivers whose children had a high viral load. Study staff hypothesized that this was due to heightened awareness of protecting immunocompromised children from getting sick during the SARS-CoV-2 pandemic (i.e., their adherence to ART may have improved when COVID infections were common, and families were home during lockdowns).

**Fig. 1:**
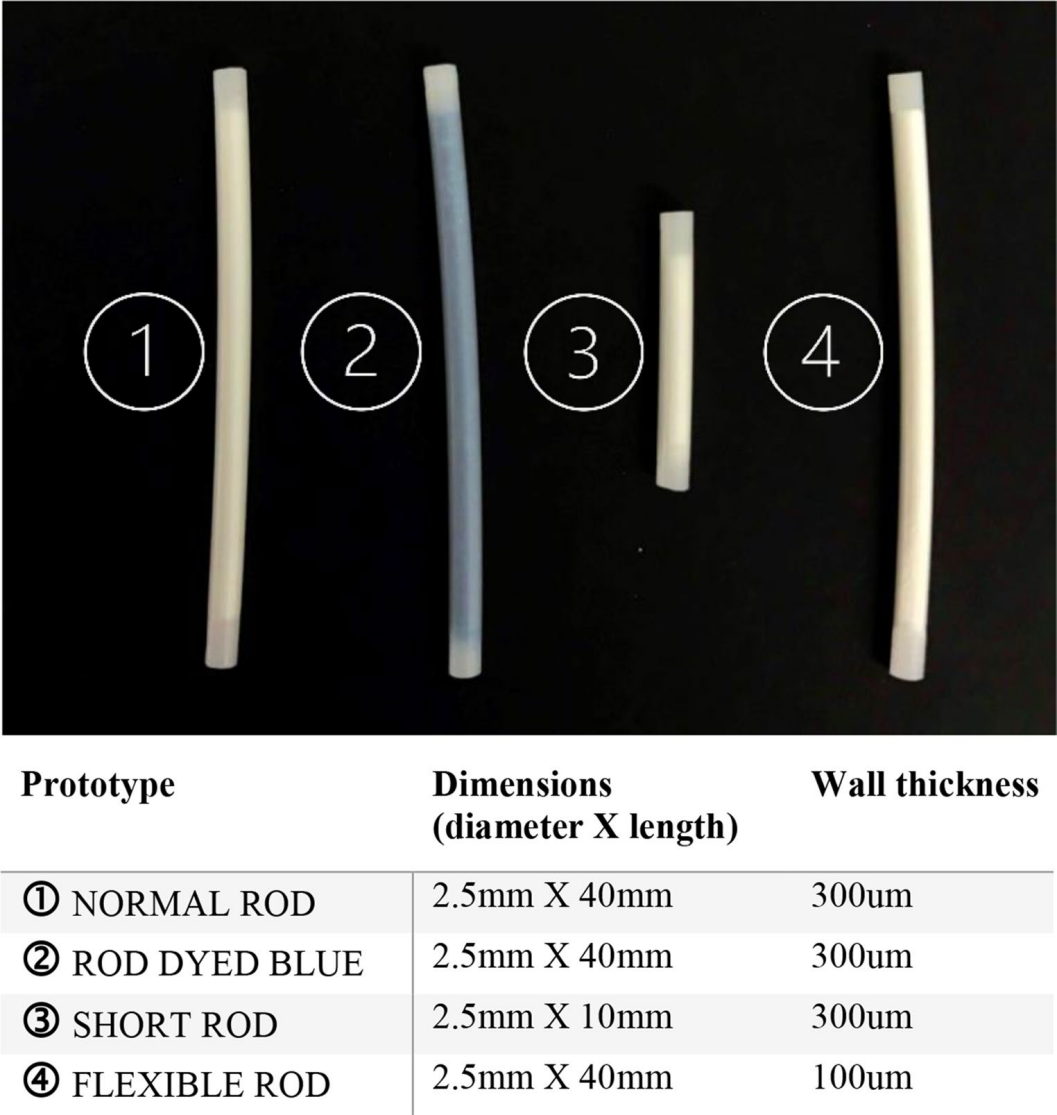
Implant prototype

The IDIs and FGDs were conducted in English, isiZulu, or Sesotho by trained social scientists in a private location at the Shandukani research site using semi-structured interview guides, audio-recorded with participants’ consent, and transcribed and translated into English. Participants were invited to handle prototypes of placebo implants varying in length, flexibility, and color during the qualitative activities. (See Fig 1) IDI topics included: caregivers’ perspectives on their child’s health and current HIV treatment, familiarity with implants, opinions on development of an implant for HIV treatment in children, product use characteristic preferences, and hypothetical willingness to accept an implant for their child’s HIV treatment. FGDs covered the same topics and explored opinions on future delivery of implants for CLWH, how implants may impact disclosure of the child’s HIV status to the child and others, and acceptability of pediatric clinical trials for a future implant. After each IDI or FGD, interviewers summarized key findings in a short, structured, debrief report. Study team members and interviewers met biweekly to discuss already collected data for possible data saturation signs and other debrief report findings.

### Data Analysis

Transcripts from the IDIs and FGDs were reviewed for quality assurance before coding and analysis, and thereafter analyzed thematically. A codebook was developed deductively using themes identified in the quality control process of transcripts and debrief reports. Two analysts used Dedoose software v9.0.62 to code an equal number of transcripts. The analysts reviewed one third of each other’s coded transcripts in full and spot-checked the remaining transcripts to evaluate intercoder reliability. They met weekly to discuss disagreements in the coding and come to consensus on code application and definitions.

Text excerpts coded with “Implant Advantages,” “Long-acting,” “Discreetness,” “Implant Drawbacks,” and “Uncertainties/Questions” were extracted based on their relevance to the research objectives of this study. The analysts wrote memoranda for each code report, summarizing findings into core concepts and then refining themes within each concept and subthemes where applicable, following a thematic analysis approach [10]. Regular meetings were held with the analysts during the coding and memoranda processes to discuss themes. Findings were then presented to representatives from the research site (including the study interviewers) to assess the validity of data interpretation and provide additional insights (See Fig 1).

## Results

### Participant Sample

This analysis included 16 IDIs and 8 FGDs with a total of 50 unique participants. IDIs included 11 biological mothers, 4 of whom had children with low viral load and 7 with high viral load, and 5 other caregivers of children with low viral load. FGDs included 4–5 participants in each, of any relationship and viral load. Data were collected from July 5th, 2021, until Feb 26th, 2022.

Most caregivers in this study were women, either biological mothers (82%) or grandmothers of CLWH (8%). Others were biological fathers, and one was a grandfather. Most (60%) caregivers had 1–2 children under their care and most participants were actively receiving an income (68%). 90% of caregivers reported living with HIV. Of those, more than half reported that they had been taking antiretrovirals for four or more years (Table 1).

**Table 1:** Participant characteristics (*n* = 50)

Characteristics	*n* (%)
Type of caregiver	
Biological mother	41 (82)
Biological father	4 (8)
Grandmother	4 (8)
Grandfather	1 (2)
Gender	
Woman	45 (90)
Man	5 (10)
Relationship status	
Single or separated	27 (54)
In a partnership or married	23 (46)
Number of children under care	
1–2	30 (60)
3–4	15 (30)
5–6	5 (10)
Ethnicity	
Ndebele	13 (26)
Sotho	9 (18)
Tswana	8 (16)
Zulu	8 (16)
Xhosa	5 (10)
Other	7 (14)
Receives an income	
Yes	34 (68)
Education	
Some secondary school or less	32 (64)
Secondary school, completed	13 (26)
Some college or university or more	5 (10)
HIV status (self-reported)	
Living with HIV (LWH)	45 (90)
Time on antiretrovirals (of those LWH)	
Less than 1 year	3 (7)
1–3 years	13 (29)
4–6 years	22 (49)
More than 6 years	7 (16)

### Findings

Current treatment options for CLWH often involve adherence challenges that impact the lives of children, their caregivers, and their interactions within broader social settings. Caregivers felt that implants as a long-acting approach to HIV treatment may offer distinct advantages compared to the current treatment options. However, caregivers also cited some apprehensions about the hypothetical implant that they felt would limit acceptability if not addressed during its development. These findings are presented in relation to three main concepts, beginning with a brief background on the current treatment challenges cited by caregivers, then describing the perceived advantages of the implant, and lastly detailing uncertainties and potential disadvantages of the implant from the caregivers’ perspectives.

### Current Treatment Challenges

Caregivers described the type of treatment their children had taken and were taking at the time of the interview, highlighting burdens of administering the medication and challenges with adherence. Children found oral medications unpalatable and often struggled to swallow pills. Caregivers explained that children sometimes refused the medicine, spat it out, or would only swallow it if mixed into food. These challenges often made children resist taking the medicine altogether and caregivers had to negotiate with them, offering incentives or enforcing adherence and creating conflict (Tables 2, 1E).

**Table 2:** Perceived advantages and disadvantages of pediatric implants for HIV treatment, by theme and sub-theme, with supporting quotes

Core Concept, Theme, sub-theme	ID #	Quote^1^
Advantages		
*Long-acting*		
Beneficial effect on viral load	1 A	Banana: I think the viral load will be stable [undetected], it will not be going up and down, it will be stable because the implant is there to give the medication in the right way. Apple: I am expecting it to drop more to a point that they will not be able to detect it, yeah.
Reduce caregiver stress over providing medication to child daily	1B	R: An implant will be better because you will not be giving the child the medication daily like you used to. Also, you will not have to be carrying the stick [stiff whip] to force her to take the medication whereas on the other hand she will be vomiting.
Would not elicit daily reminder that child is living with HIV	1 C	Pinki: As for me taking care of a child who is living with HIV is very difficult. It is very difficult for me to see a child and giving the child medication each and every time [day] because it is very hurting, you see.
Allow child to stay with family members who will not have to administer medication	1D	R: It [the implant] is most useful because even if it happens like today, I left early and I do not know if they [family members] gave the child the medications right, so, but if it [the implant] is inserted in him [the child], I know for sure he [the child] is okay.
Avoid the need for disciplining child when they don’t want to take medication (relationship and caregiver fatigue)	1E	Survivor: I want this thing [HIV] stopped in his body because the time when he had blisters, he stressed me too much, he stressed me so bad. And this thing of taking tablets when my son takes tablets so every time when I’m giving him tablets, I have to carry a belt. Mabree: To beat him.
*Discreetness*		
Prevent child from wondering why they are taking medication every day unlike other kids	2 A	Strawberry: She [the child] sometimes gets naughty because some time, which is why I am saying I wish the implant were available now, because she often sits me down and ask, “Until when I am going to take these tablets?” You see.
Allows caregiver and child to keep status secret from others	2B	Jowie: Again, it [the implant] will help with the issue of confidentiality, I do not have to worry about who else will know that the child is taking [HIV] medication because we have those issues. It will be secretive under the skin, now there is no more worries to say when you are visiting what is going to happen.
Minimizes experiences of stigma and discrimination from others	2 C	Mpho: Some of us don’t like being asked questions by strangers, so it’s just questions, silly questions so that’s why we want it [the implant] to be invisible… [People would ask] “What is this thing? Is this that thing for sick children? Are you also sick?” Yeah, those are the questions they would ask.
Compatible with traditional medicines and customs	2D	Jowie: Here, especially when you are dealing with something new there is always questions marks, but with this one [implant] inserted, but culturally there is a belief they call *ukugcaba* [a custom of making small incisions in the skin to insert traditional medicine], or applying something topically, that thing stays [in the skin] permanently. Everybody can see that you have scars, so it will not be that bad, it becomes normal.
***Disadvantages***		
*Efficacy*		
Worries about whether effective compared to current options and when used with traditional herbs	3 A	Apple: My husband used to take *imbiza* [a concoction of traditional medicines] to cleanse his stomach. Now he cannot anymore, and it is really frustrating for him because we were advised that he must take something light like ‘Mist Alba’ [commercial laxative] and he does not like it. Not because it is bitter but because it does not do what he wants as compared to a traditional concoction. Apparently with *imbiza*, he was feeling that it was effective in cleansing his stomach, [but] with Mist Alba, he feels like he needs to use it, like, every month.
Unacceptable if it does not lower viral load	3B	Scorpion: I think even now, like my son is taking the tablets, like it [viral load] is not stable. But then if the difference is big, like [at] every appointment it [the viral load] is abnormal, like either too high or, then there’s a problem. Then I would suggest taking him [the child] [go] back to the tablets.
*Safety/Side Effects*		
Worries about unacceptable side effects, e.g., infertility, nausea, allergic reactions	4 A	Tea: I would say, like, I have a girl child, fertility is everything for a woman. So, for my child I would say it is the main pivotal point for me, if it [the implant] is going to affect her womanhood, yeah…
Concerns that the implant might be unsafe to use with other medications	4B	Apple: But now with the other medication [multivitamin] they [healthcare workers] still give us, do we still give that medication, or do we stop when it comes to [HIV] virus and treat whatever that the child has at that moment?
Preferences for implants that are not colored and can be removed early if needed	4 C	Pinki: Transparent [implant] is better because other colors, we do not know what damage they can cause if the [colors] come out.
*Pain*		
Insertion and removal procedure may be painful for the small child	5 A	Tea: I know personally it was painful as my hand was painful for two weeks [after inserting the contraceptive implant]. So, like, in terms of pain, how do you overcome it as doctors and all that?
Concern that the implant may move/shift while the child is playing and cause harm	5B	Precious: I think it [flexible implant] is okay so that the children cannot feel pain. If it bends it cannot hurt them, it is going to be easy and sit nicely.
Child might play with implant under skin if it is not flexible or placed somewhere discreet	5 C	R: Children can be naughty, they are not like adults, with adults if it is sitting there, they [adults] will not play with it.

^1^ Quotes are labelled with pseudonyms chosen by participants in FGD, or with “R” for “respondent” in an IDI

Caregivers also explained that some current medications like syrups were physically cumbersome, stored in glass bottles and necessitated administration via syringe, which was not often on hand. Many caregivers noted that oral medications required frequent collection from clinics, which added to their responsibility and required time away from work.

Overall, caregivers liked the idea of the pediatric HIV treatment implant and in principle felt that it was preferrable to the current treatment options. One participant was quoted saying, *“Lord*,* let there be at least a way [of administering HIV treatment] for children that is simpler. Because I have seen with my child… because no child asks for this [HIV positive status].”* The themes that emerged in discussions about implant advantages and disadvantages are presented in more detail in the sections that follow.

### Implant Advantages

The advantages of the implant discussed among caregivers were categorized into two major themes during coding and analysis: long-acting duration and discreetness.

Given the challenges that caregivers face when physically administering medication to their children, many highlighted that a long-acting product like an implant would reduce the frequency of this burdensome experience. Further, caregivers remarked that the longer duration of an implant could improve the child’s health by supporting sustained adherence to ARVs (Tables 2, 1A). Challenges of adhering to daily oral treatment mentioned by caregivers included conflicting schedules with work or school, forgetting to give medication, mishandling medications (e.g., spilling syrup), and children struggling to take the medication (e.g., spitting it out) (Tables 2, 1B). Caregivers also noted that the availability of longer-acting medication might relieve some of their stress related to caring for a child living with HIV. Stressors they cited included: the need to keep medication hidden from others, fear of impacting their child’s health by forgetting or defaulting on medication, and the need to visit clinics frequently to pick up medications, which in turn resulted in missed work and financial stress from transportation costs.

A few caregivers noted that the long-acting implant would eliminate the daily reminder that the child is living with HIV, which creates feelings of guilt and/or shame (Tables 2, 1C). They also explained that some children experience stress or confusion about the requirement to take medication every day, creating the potential for caregiver-child conflict when children asked about their medicine and caregivers felt the need to tell a ‘white lie’ to not disclose their HIV status. For example, caregivers would tell them that the medication treats allergies. Many caregivers felt that an implant could minimize this conflict by reducing questioning from children about the purpose of the medication (Tables 2, 2A). A participant stated, *“She [the child] sometimes gets naughty because some time*,* which is why I am saying I wish the implant were available now*,* because she often sits me down and ask*,* “Until when I am going to take these tablets?” You see.”* The long-acting and discreet nature of the implant were also seen as features that would allow their child to stay with family members who were not yet aware of the child’s HIV status (Tables 2, 2B). A participant stated, *“Again*,* it [the implant] will help with the issue of confidentiality*,* I do not have to worry about who else will know that the child is taking [HIV] medication because we have those issues. It will be secretive under the skin*,* now there is no more worries to say when you are visiting what is going to happen.”* Children using implants would also be more easily supervised by others, such as teachers, neighbors or child-minders, for longer periods of time, as there would be no need for medication administration by the caregiver at particular times of the day. Discreetness, in short, was seen as an element that may minimize experiences of stigma and discrimination by allowing the child’s status - and even that of the caregiver – to remain private (Tables 2 and 2C).

Caregivers cited various other advantages of the implant compared to oral medications, offering perspectives about how an implant would mitigate or minimize burdens they currently face. They discussed how the implant would reduce their need for disciplining and even punishing their child when they didn’t want to take medication. A few caregivers mentioned physically disciplining their child with a stick or belt when the child resisted taking medicine (Tables 2, 1B & 1E). A participant stated, *“An implant will be better because you will not be giving the child the medication daily like you used to. Also*,* you will not have to be carrying the stick [stiff whip] to force her to take the medication whereas on the other hand she will be vomiting.”* Some also noted that an implant may be more compatible with traditional medicines and healing practices, as compared to current oral medications. For example, the use of traditional herbs or enemas were seen as incompatible with the taking of oral medications because they impair drug absorption. One participant also mentioned a local practice of inserting traditional medicines into shallow incisions on the skin, which they felt could make the implant more acceptable to caregivers or mean that scars from an implant would not stand out as necessarily unusual (Tables 2 and 2D).

### Implant Disadvantages

The discussions with caregivers also explored their worries and uncertainties about the implant. Themes relating to implant disadvantages were grouped into concerns or uncertainties about efficacy, safety or side effects, and pain.

Some wondered about the efficacy of the implant as compared to current treatment options and when used together with traditional herbs (Tables 2, 3A). Participants said some members of their community hold apprehensions about new products, preferring the use of herbal mixtures or medicines instead that they deem as more effective. Caregivers also all agreed that the implant would be unacceptable if it failed to lower children’s viral loads compared to current treatments (Tables 2, 3B).

Safety and side effects were raised as concerns that could impact the acceptability of the implant, depending on their severity. Caregivers cited various potential side effects, the most salient of which included concerns about infertility, nausea, and allergic reactions (Tables 2, 4A). Another specific concern of some participants was related to blood circulation or toxicity and the potential biodegradability of the implant. A participant stated, *“Looking at the facts if it [the implant] dissolves it becomes liquid and blood is liquid*,* what if it fuses with the blood and causes some [side effects].”* Concerns also emerged that the implant might be unsafe to use concurrently with vitamin supplements and with other current medications (Tables 2, 4B).

The worry over side effects influenced caregivers’ preferences for product characteristics, such as whether the implant should be an opaque color or transparent. Participants were uncertain about an opaque product because of worries that a colorant could “seep” out and create health problems like infections or skin cancer or alter the color of the child’s urine (Tables 2, 4C). Owing to these concerns, they also preferred the idea of an implant that could be removed early, if needed, and felt that the implant would be unacceptable otherwise.

The perceived pain that the child might endure during implant administration, use, and removal was also a topic of concern among some caregivers (Tables 2, 5A). Some related their own experience with the contraceptive implant when worrying about the pain for the child, particularly during the insertion and removal process. One participant stated, *“I know personally it was painful as my hand was painful for two weeks [after insertion of the contraceptive implant]. So*,* like*,* in terms of pain*,* how do you overcome it as doctors and all that?”* There was also concern that the implant may move subcutaneously while the child is playing and cause harm or pain (Tables 2 and 5B). If movement of the implant were to occur, some wondered if the implant would remain viable and effective. The participants’ concern regarding their children’s freedom of movement also influenced their preferred characteristics, favoring a more flexible implant due to concerns that a stiff implant would hurt the child more. Some also worried that children may tamper with an implant under the skin if not placed discreetly, potentially causing harm (Tables 2, 5C).

## Discussion

In this study, we aimed to understand the acceptability of an implant for pediatric HIV treatment from key decision-makers and stakeholders of CLWH’s care. Implant specifications, including their physical shape and design, drug composition and release, and biodegradation profiles are under investigation. These findings are valuable to guide the product development and align with the needs of caregivers of CLWH. In addition to modifiable features of the implant design, overall contextual attitudes towards the technology are imperative for pipeline development. Importantly, this sample of caregivers of CLWH corroborated the challenges with current treatment options found in earlier research [11, 12], and expressed a definitive need for alternative approaches to pediatric HIV treatment. They readily saw the potential benefits of a long-acting approach enabled by an implant, and discussed several key characteristics that centered around: (1) the overall acceptability of the implant for caregivers as decision-makers for CLWH’s care; (2) the balance of implant advantages versus disadvantages, as perceived by caregivers; and (3) caregivers’ potential uncertainties and concerns about the implant to be addressed during product development, testing, and delivery.

The general sentiment from caregivers in this sample was that a long-acting implant would be an acceptable platform for HIV treatment in young children. Challenges with current treatment options described above are barriers to adherence and, relatedly, maintenance of viral suppression in children. Many caregivers expressed a strong opinion that an implant would help address those barriers to adherence and ultimately benefit CLWH’s health and wellbeing. Importantly, concerns expressed by caregivers about the safety of the implant were not as salient as healthcare providers had anticipated from caregivers in this setting [11, 12]. This highlights the importance of conducting research among primary end-users – caregivers in this case – rather than relying on opinions of secondary-level informants or other stakeholders to anticipate or predict many primary end-users’ opinions. The importance and prerequisite for end-user input in the acceptability of new biomedical products has been voiced in other research, including studies on implants for HIV prevention and treatment [11–18]. These findings suggest there is strong overall support among end-users to continue research and development of an implant to help overcome some of the experiential challenges with current pediatric treatment options.

Analysis of these data showed that the implant advantages identified by the caregivers potentially affected a much wider range of stakeholders than the disadvantages. Advantages of the implant as cited by caregivers were linked to multiple human factors, including positive impacts on the health of the child, the wellbeing of the caregiver, the relationship between the caregiver and child, and dynamics with extended family and community members. Perceived disadvantages, on the other hand, centered primarily around a possible negative impact on the health of the child alone. These included efficacy, safety, and side effect concerns. These findings are reminiscent of how some pregnant and breastfeeding women who used longer-acting drug delivery platforms (e.g., monthly vaginal rings) for delivery of HIV prevention drugs (e.g., dapivirine) expressed some concerns about longer-term health impacts on their infants [19, 20]. While a negative health impact on the child undoubtedly holds far-reaching repercussions on caregivers, family, and health systems, caregivers in this sample did not mention those possible ripple effects.

Indeed, the caregivers’ focus on the possible positive repercussions across these various socioecological levels, combined with literature underscoring the challenges to pediatric adherence and viral suppression in this endemic setting [1, 2, 6], suggests a substantial need for a long-acting and discreet approach that would alter the current structure of CLWH’s care. As such, the potential advantages of the implant platform could provide added benefits by addressing larger structural barriers and challenges of children’s HIV treatment, care, and livelihood. In addition, the child-focused disadvantages caregivers cited were solvable and would be addressed in the future during implant development and phased human testing. For example, the concerns about pain tolerance may be addressed with provider training on insertion and removal, as well as anesthetic administration. Safety, efficacy, and side effects would be closely monitored throughout the clinical testing phases of product development, with safety and regulatory oversight by Data Safety Monitoring Boards and IRBs. In other words, the clinical trial process would, by definition, prohibit the implant from moving forward in the development pipeline, if these critical components of adequate safety and efficacy that were expressed as concerns by caregivers, had not been addressed.

There are limited data about the use of implants with young children who may present unique challenges and needs given their small size, high activity levels, and anatomical development. For example, CLWH may have low adipose tissue around implant insertion sites. It will be critical to continue to evaluate the acceptability of an implant in this population during safety and efficacy trials. Irrespective of whether a safe, effective, long-acting, biodegradable implant is the most commonly preferred HIV treatment option, an alternative treatment choice is critical for those who find daily oral medications challenging or incompatible with their lives. Further, a choice in treatment options for children may have positive secondary mental and physical health effects on caregivers, who otherwise struggle with maintaining their children’s treatment.

Our research has several limitations. The recruitment plan for caregivers was not developed to garner a representative sample of all caregivers of CLWH in South Africa, and instead was based on access to the population of interest at public sector clinics in the surrounding area of the research site. The recruitment plan originally planned to include caregivers of children with a high viral load. During recruitment efforts, however, we encountered challenges finding such caregivers. We hypothesized that children’s HIV viral loads had improved during the COVID pandemic because caregivers had elevated attention towards ensuring their child’s immune system was not compromised. Caregivers were also at home more often during the pandemic, potentially enabling a stricter schedule of administering medication. We also limited the scope of our inquiry about long-acting alternative treatment options to the implant and not to other delivery platforms (e.g., injections, patches, etc.) because our objective was to provide pipeline bridging feedback on preferred user characteristics and acceptability of the implant to the product development team. Further research is required to explore the acceptability and preferences of the implant in comparison to other pediatric HIV treatment options among caregivers.

## Conclusion

Caregivers in this study indicated that implants would be an advantageous treatment option for their CLWH and recognized that implants may provide ancillary benefits to their everyday lives, such as improving family dynamics, providing the opportunity for others to care for the child, decreasing caregivers’ stressors, and potentially minimizing children and caregivers’ experiences of stigma and discrimination. While implants are a less familiar treatment option for CLWH than tablets and syrups, caregivers voiced a desire for an option that is long-acting for easier adherence. Further pre-clinical and clinical development and testing, combined with mechanisms for ongoing end-user input is warranted as an important option for pediatric HIV treatment.

## Data Availability

Due to the sensitive nature of and potential indirect identifiable information in qualitative research, supporting data is not available.
